# Association of 340B entity eligibility with changes in hospital financial performance

**DOI:** 10.1093/haschl/qxag147

**Published:** 2026-06-10

**Authors:** Neal Masia, Jonathan D Campbell, Yevgeniy Feyman, Kenneth Finegold

**Affiliations:** Department of Economics, Columbia University, NewYork, NY 10027, United States; Health Capital Group LLC, Aventura, FL 33160, United States; Research Department, National Pharmaceutical Council, Washington, DC 20006, United States; Research Department, National Pharmaceutical Council, Washington, DC 20006, United States; Research Department, National Pharmaceutical Council, Washington, DC 20006, United States

**Keywords:** 340B drug pricing program, hospital finances, health policy research, Affordable Care Act (ACA)

## Abstract

**Background:**

The 340B program requires pharmaceutical manufacturers to provide discounts on drug purchases to hospitals caring for a large share of vulnerable populations. Recent concerns raised by legislators and regulators have highlighted potential misuse of revenue from the program. Thus, we explored changes in the financial performance of 340B-eligible hospitals.

**Methods:**

Focusing on 340B-eligible hospitals (excluding critical access hospitals), we used 2023 data from Medicare Cost Reports and the Office of Pharmacy Affairs Information System to identify changes in several measures of financial performance among 340B and non-340B hospitals. Descriptive statistics and linear regressions were used to estimate the magnitude of associations.

**Results:**

The average 340B hospital was associated with 47.5% (95% CI: 36.7%-58.3%) greater total assets. The number of 340B sites was associated with higher real asset growth, with 10 additional 340B sites associated with a 0.15% (95% CI: 0.05%-0.25%) increase in the real asset growth rate. 340B hospitals also exhibited higher spending on administrative salaries than non-340B hospitals.

**Conclusion:**

These findings suggest that 340B-eligible hospitals have had consistently strong financial performance, surpassing non-340B-eligible hospitals. The results provide insight into the ongoing policy debates surrounding how 340B hospitals use proceeds from the program.

## Introduction

The 340B drug pricing program (340B) was created by Congress in 1992 to ameliorate the unintended consequences of the best price provisions of the Medicaid Drug Rebate Program (MDRP). After enactment of MDRP in 1990, pharmaceutical manufacturers limited the discounts uniquely available to safety-net hospitals.^1^ Under the terms of the 340B program, drug manufacturers participating in Medicaid and Medicare Part B are required to provide steep discounts on covered outpatient drugs to 340B covered entities (CEs). According to the Health Resources and Services Administration (HRSA), which oversees 340B, the purpose of the program is to “stretch scarce federal resources as far as possible.”^2^ CEs may sell (or give away) 340B outpatient drugs to patients of the CE either directly or through contract pharmacy arrangements. CEs can earn profits on the spread between the reimbursed price of each drug and the 340B acquisition costs, a gap that varies between roughly 23% and 99%.^3^ CEs purchased over $81.4 billion in discounted 340B drugs in 2024,^4^ and those drugs are estimated to have generated over $147 billion in sales.^5^

340B has grown rapidly due to significant policy and regulatory changes since the enactment of the Affordable Care Act (ACA). Specifically, the number of 340B CEs and child sites (eg, outpatient clinics and treatment centers associated with the covered entity) has grown steadily as new categories of eligibility and rules regarding existing categories have expanded.^6^ There are now thousands of CEs, contract pharmacies, and child sites, often in higher-income areas full of well-insured patients.^7,8^

Research on 340B has generally found evidence of unintended consequences.^9^ Though one study has found evidence of lower drug prices charged to commercial insurers by 340B-eligible facilities,^10^ other work has demonstrated that greater local 340B activity is associated with higher overall Medicaid spending,^11^ higher employer insurance premiums,^12^ and higher ACA premiums,^13^ and 340B program eligibility has been associated with hospital acquisition of physician practices.^14^ Hospitals in more consolidated systems are also more likely to join and profit from the 340B program.^15^ Evidence also suggests that 340B hospitals generate higher operating profits, offer less charity care, and charge higher commercial prices.^16-19^ Such studies are inherently limited because they focus on a point in time; no research to date has examined how longer-term participation in the 340B program is associated with hospital finances or operating practices.

Given recent interest in understanding the profits generated by 340B covered entities and the extent to which those profits are shared with patients,^20,21^ we sought to understand how changes in hospital finances over time were related to 340B program participation.

## Data and methods

### Data: outcomes

We focused on the accumulation and level of overall financial assets as a key metric of hospital health and performance over time, comparing the experiences of 340B and non-340B hospitals. We collected hospital data for 2013-2023 using Medicare cost report data (cleaned and filtered by RAND).^22^ Cost report data are reported by every Medicare-certified US hospital annually. Recent research has found that hospitals with faster-growing assets also have higher rates of executive compensation,^23^ so we also gathered administrative salary data to test whether 340B participation is associated with a differential in administrative salaries.

We studied 3 key metrics calculated from measures reported directly on the cost reports:

1. Total assets, defined in the cost reports and including cash, investments, and other assets.^24^
2. Real asset growth 2013-2023, defined as the compound annual growth rate (CAGR) of the hospital's total reported financial assets, after adjusting for inflation based on the Consumer Price Index for All Urban Consumers (CPI-U).
3. Administrative salary spending, which is captured from the cost report Worksheet A, Line 5, Column 1 (the salary subcomponent of the overall “administrative and general” cost center).

We converted the level of total assets and level of administrative salaries variables to natural logarithms so that the coefficients can be interpreted as percentage changes in the dependent variable of interest. Taking the natural logarithm did not affect observations as no observations had zero or negative values. None of the variables used had missing values.

### Data: predictors

We used data from the HRSA Office of Pharmacy Affairs Information Systems (OPAIS) database to identify 340B participation by year and identify active child-site and contract pharmacy relationships associated with each hospital for each year. Participation in 340B was operationalized as an annual variable taking the value 0 or 1. The number of active child-sites and the number of contract pharmacy relationships were operationalized as the counts of these values.

### Sample exclusions

Our focus was on nonprofit community hospitals, including privately-owned and government-owned hospitals. The most common path to 340B eligibility is to achieve a disproportionate share (DSH) adjustment of at least 11.75%. The DSH adjustment percentage captures the share of low-income patients treated at the hospital and depends in large part on the share of inpatient days for patients covered under Medicaid.

Critical access hospitals (CAHs) are small (under 25 hospital beds) rural hospitals that typically have more flexible reporting requirements than other hospitals and are automatically eligible for 340B. For these reasons and consistent with prior work, we exclude CAHs from our analysis.^14^ There are other, less frequent types of 340B hospitals with different eligibility rules, including children's hospitals, cancer hospitals, and rural referral centers. These hospitals were included in the analysis.

### Statistical analysis

We estimated 3 separate linear regressions with the outcomes listed previously. The regressions are cross-sectional and are at the hospital level, measuring changes in the variables from 2013 to 2023. To explain variation in hospital financial performance, we included the following variables from the cost reports as covariates: the number of inpatient beds, rural/urban location, status as a major teaching hospital (Defined as per the Medicare Cost Report, typically with an intern and resident to bed ratio of 0.25 and/or membership in the Council of Teaching Hospitals), operating revenue, and operating profit margin. These variables have previously been used in assessing hospital outcomes and performance (Ibid). To assess the relationship between 340B status and financial performance, we included an indicator for whether the hospital was a 340B program participant in 2023, and variables measuring the number of 340B sites operated by the hospital, the number of contract pharmacy relationships, and the number of years the hospital has participated in the program.

Because of variation in state-level policies (such as bans on for-profit hospital ownership and different Medicaid eligibility criteria) that may affect hospital 340B eligibility, we also included state fixed effects in the linear regressions. This compares hospitals in a state with other hospitals in the same state, and accounts for unobserved heterogeneity between states. We used heteroscedasticity-robust standard errors.

### Interpretation

We captured the mean values for the 3 dependent variables in our regression—total assets, asset growth rates, and administrative salaries—for the DSH hospitals in our sample, and calculated the impact of the statistically significant 340B variables from each regression on the relevant variable.

## Results

Our analytic sample included 2605 community hospitals with complete cost reports over the time period, 45.8% (1194) of which were 340B hospitals. Most 340B hospitals (86.2%) were DSH-eligible. Summary statistics on the hospitals in our analytic sample and relevant subcategories are shown in Table 1. Real (inflation-adjusted using CPI-U) assets grew at an average of 1.2% annually for the hospitals in our sample. Overall, assets grew slightly faster at 340B hospitals (1.4% per year), while 340B DSH hospitals grew at 1.5% per year. Non-340B hospitals grew assets at 1.0% per year on average. The average DSH hospital had total assets of $831 million in 2023, or $3.7 million per bed, somewhat higher than the average of $2.7 million per bed for all nonprofit hospitals and substantially higher than the $2 million per bed figure for non-340B hospitals.

**Table 1:** Key data characteristics, 2023.

	All hospitals	All hospitals excluding CAH	All 340B hospitals excluding CAH	340B DSH hospitals
*Number of hospitals in dataset*	*3621*	*2605*	*1194*	*1029*
Mean bed size	149	199	264	279
Mean operating revenue (millions)	$308	$410	$626	$655
Mean CPI-adjusted asset CAGR, 2013-2023	1.8%	1.2%	1.4%	1.5%
Mean total assets (millions)	$395	$529	$811	$831
Mean total fund balance (millions)	$236	$316	$432	$428
Mean days cash on hand	115	89	115	110
Mean administrative salaries (millions)	$11.5	$15.2	$24.8	$25.1

Source: Author analysis of data from Medicare Cost Reports as tabulated in RAND Hospital data sets, available at hospitaldatasets.org.

Regression results are shown in Table 2. We found that 340B participation, teaching status, the number of beds, operating profits, and revenues were all associated with higher total asset levels, while rural status had a negative relationship with total assets after controlling for the other factors. Specifically, 340B participants had 47.5% (95% CI: 36.7%-58.3%) greater total assets, with smaller associations among other covariates. We found that the number of 340B sites, teaching status, operating revenue, and operating margins were each associated with higher real asset growth. Our results suggest that 10 additional 340B sites are associated with a 0.15% (95% CI: 0.05%-0.25%) increase in annual real asset growth over the period.

**Table 2:** Fixed effects regression results for all non-CAH hospitals.

	Real asset growth rate, 2013-2023		ln (Total assets), 2023		ln (Administrative salaries), 2023	
	Coeff.	*t*-stat	Coeff.	*t*-stat	Coeff.	*t*-stat
**340B sites**	**0**.**000149**	**2**.**91**	−0.00209	−1.52	−0.0013	−1.80
**RX contracts**	−0.000047	−1.31	−0.00003	−0.05	0.0009	1.95
**340B participant**	0.000211	0.05	**0**.**47477**	**8**.**64**	**0**.**2275**	**5**.**15**
**Years in 340B**	0.000342	1.30	0.00528	1.51	**0**.**0122**	**4**.**18**
**Rural**	−0.004486	−0.95	**−0**.**30566**	**−4**.**21**	**−0**.**2032**	**−5**.**59**
**Beds**	−0.000024	−1.58	**0**.**00315**	**9**.**25**	**0**.**0023**	**16**.**84**
**Teaching**	**0**.**016019**	**3**.**48**	**0**.**33243**	**4**.**60**	**0**.**2433**	**4**.**54**
**Operating rev (mil)**	**0**.**000010**	**2**.**58**	**0**.**00025**	**2**.**13**	**0**.**0003**	**6**.**56**
**Operating margin**	**0**.**104173**	**8**.**21**	**1**.**31326**	**7**.**63**	**0**.**5181**	**4**.**41**
***N***	2603		2603		2602	
***R* ^2^**	0.12		0.62		0.63	

All regressions include state-level fixed effects and robust standard errors. Bolded values indicate statistical significance at *P* < 0.05.

Source: Author analysis.

Our results also show that 340B hospitals with higher revenues, higher profits, teaching status, and more beds spend more on administrative salaries, while rural hospitals spend less. Notably, even after accounting for these factors, 340B participants spent 22.8% (95% CI: 14.1%-31.4%) more on administrative salaries than nonparticipants.

## Discussion

From 2013 to 2023, we found that the average 340B DSH hospital had stronger rates of asset accumulation than non-340B hospitals. DSH hospital assets grew by 1.5% per year, and 340B participation contributed to a 47.5% increase in assets compared to non-340B hospitals. Our findings on 340B hospital asset growth are consistent with recent research that found substantial asset growth in wealthy hospitals over time.^16^ Table 3 provides the necessary context to interpret the impact of 340B on the variables in question by applying the results to the average DSH hospital. Our findings suggest that 340B hospitals were associated with $276 million higher assets on average. Applying the average to the 1029 DSH hospitals in our data set, we estimate that DSH hospitals accumulated $284 billion in total assets associated with 340B as of 2023. Finally, our findings suggest an association between increased administrative salaries and 340B DSH hospitals—translating to approximately $7.0 million more per year for the average 340B DSH hospital in our sample.

**Table 3:** Impact of statistically significant (*P* < .05) 340B-related factors for the average DSH hospital (*n* = 1029).

	Mean value—all DSH hospitals	Real asset CAGR 2013-2023	Total assets (millions)	Admin salaries (millions)
**Mean—all DSH hospitals**		1.54%	$831	$25.1
**340B sites**	21.4	0.32%	n/a	n/a
**340B participant**	1	n/a	+47.4%	+22.7%
**Years in 340B**	12.9	n/a	n/a	+15.7%
**Total impact**		+0.32%	+47.4%	+38.4%
**340B impact**		+20.1%	+$276 million	+$7.0 million

Source: Author analysis.

Our observations that 340B hospitals have accumulated more assets than non-340B hospitals are aligned with a report from the Minnesota Department of Health, which found that the largest 340B DSH hospitals financially benefited the most from the program, representing over 80%, or more than $1 billion, of statewide 340B revenue.^15^ Similarly, the U.S. Senate Committee on Health, Education, Labor, and Pensions investigated 2 large 340B health systems and found that they generated hundreds of millions of dollars in savings and revenue from 2018 to 2023.^14^

Insurance prices, outpatient procedure costs, and drug costs have also been investigated at 340B hospitals. Previous research found that the prices large 340B hospitals charge commercially insured patients are, on average, 7.5% higher than those of the same-sized non340B hospitals, and these 340B hospitals bill 24% more for outpatient procedures.^25^ This finding is consistent with a more recent white paper that analyzed drug cost per outpatient hospital patient across commercial, Medicare Advantage, and Medicare Fee-For-Service coverage. Drug cost per outpatient hospital patient in 2023 was approximately 3 times higher at 340B DSH hospitals than at non-340B DSH hospitals.^26^ Evidence suggests that 340B participation has contributed to hospital-provider consolidation^10^ and horizontal hospital consolidation,^27^ and that both types of consolidation have been shown to raise prices for many healthcare services.^28^ Additionally, access to 340B prices impacts decisions made around the site of care for oncology patients (which was shown to increase Medicare spending by over $1100 per patient).^29^ One study found that 340B hospitals received markups on outpatient drugs that were 6.59 times those of independent physician practices.^30^ Consistent with these findings, peer-reviewed research shows that higher levels of 340B activity lead to higher premiums for ACA plans,^9^ and other evidence has shown that employer-based insurance premiums increase with 340B activity at the state level after controlling for other factors.^31^ Taken together, these findings present a consistent picture suggesting that hospitals generate substantial profits from 340B, and that the program influences their behavior and serves as an important cost driver across the broader ecosystem.

## Conclusion

The 340B program, intended to help vulnerable Americans, has grown rapidly in recent years. Our results suggest that the 340B program is associated with increased hospital assets and administrative salaries. Nonprofit hospitals have garnered significant attention for their strong financial performance. Our findings provide evidence that this is at least in part due to the benefits of the 340B program, suggesting a need for reform.

## Supplementary Material

qxag147_Supplementary_Data

## Data Availability

RAND Hospital Cost Report data cannot be shared under the data use agreement.
